# Anchorage performance of a high-pressure pre-tightening resin anchor with a compressed grouting body

**DOI:** 10.1371/journal.pone.0171653

**Published:** 2017-02-14

**Authors:** Jiansheng Tian, Li Hu

**Affiliations:** 1 School of Mechanics & Civil Engineering, China University of Mining and Technology, Xuzhou, China; 2 PowerChina Roadbridge Group Co., Ltd., Beijing, China; Massachusetts Institute of Technology, UNITED STATES

## Abstract

Supports for deep mine roadways located in soft surrounding rock face several problems: difficulty in applying pre-tightening force, low bearing capacity, and poor initial support. To solve these problems, this study proposes a high-pressure pre-tightening resin anchor with a compressed grouting body for use in soft and fractured rock surrounding a deep roadway. Using model experiments, we analyzed the anchorage performance of the proposed anchor and a conventional tensile-type anchor for three different values of the elastic modulus of the surrounding rock. The results showed that regardless of the surrounding rock type, the peak micro-strain (642–541) and displacement (6.09–6.5 mm) at the pull-out end of the proposed anchor were always smaller than the peak micro-strain (1433–1105) and displacement (8.77–9.2 mm) at the pull-out end of the conventional anchor. Furthermore, as the anchor’s pre-tightening force increased from 20 kN to 120 kN, the anchor’s strain remained concentrated over a length of 0.4 m from the bearing end. Compared with conventional tensile-type anchors, the proposed high-pressure pre-tightening resin anchor with a compressed grouting body has a higher ultimate bearing capacity, allows the grouting length to be decreased to 0.4 m, and provides initial support resistance.

## Introduction

As a rule, coal mining proceeds from a shallow stratum to a deep stratum. When deep roadways are used for mining, the crustal stress significantly increases with increasing mining disturbance intensity. Additionally, the surrounding rock is often soft rock composed of sandstone, mudstone, and sandy mudstone, which is low in strength [1–4]. Therefore, the surrounding rock undergoes relatively large deformations during the initial excavation period, resulting in cracking damage to the roadway cross section. To effectively control the deformation damage to the surrounding rock during the initial stage and to ensure coal mining safety, a secure and reliable support method must be provided for the soft and fractured surrounding rock environments of deep roadways.

**Fig 1:**
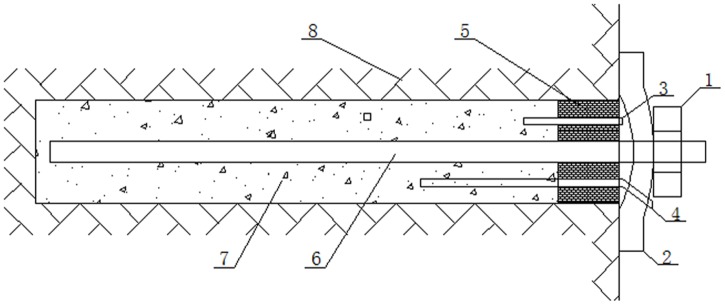
Schematic diagram of the anchorage structure for a grouting anchor: 1-nuts, 2-anchor tray, 3-exhaust pipe, 4-grouting pipe, 5-sealing plug, 6-anchor, 7-grouting body, and 8-surrounding rock.

Pre-tightening force anchors are most often used to provide support for deep roadway anchorage [1]. Practical experience has indicated that whether the anchors in the surrounding rock of a deep roadway can sustain sufficient pre-tightening force is crucial for the realization of active support, effective control of the deformation of the rock, and the quality of support that the anchors provide for the roadway [2–5]. Grouting anchors (also called tensile-type anchors) have recently been used to support the fractured rock surrounding deep roadways. Grouting anchorage can effectively improve the integrity of the rock surrounding a roadway and can also improve the cohesive force and internal friction angle [6–8]. The self-stability of the surrounding rock can thus be improved along with the support for and quality of the roadway. The anchorage structure of a grouting anchor is shown in Fig 1. The bearing mechanism of a conventional grouting anchor is as follows: a torque is applied to the anchor nuts, which is then transferred to the anchor, and the anchor sustains tension, which is applied directly to the grouting body through the anchor. As a result, under tension, the grouting body plays a role in anchoring the surrounding rock.

However, the free length of a grouting anchor is always quite short, which greatly limits its pre-tightening force [9,10] and prevents the anchor from achieving active support. Before a pre-tightening force can be applied to the anchor, the grouting body must first be cured and allowed to reach a certain strength. This process generally lasts 7 days, and this length of time is a severe disadvantage for deep roadways, which require timely and sufficiently strong support during the initial excavation stage. In addition, after the application of the pre-tightening force, the grouting body is bonded to the anchor but is in an unfavorable stressed state; in this state, the brittle grouting body is in tension [11–14]. Therefore, the pre-tightening force that can be applied is rather small, and the anchorage in the surrounding rock is relatively weak. This weak anchorage can further decrease the support provided by the grouting anchor or even cause the anchor to fail.

Therefore, a grouting anchor (specifically, a tensile-type anchor) or a resin anchor cannot achieve the support required for both a high pre-tightening force and rapid initial support while simultaneously filling in the crevices in soft and fractured surrounding rock [13,15–17]. To overcome these deficiencies and to improve the design of supports for deep roadways in soft surrounding rock, this study proposes a new type of anchor supporting method that is suitable for soft and cracked surrounding rock: a high-pressure pre-tightening resin anchor with a compressed grouting body. Such anchors can effectively solve the problems associated with conventional supports for deep roadways in soft surrounding rock, namely, their poor initial strength, the difficulty of applying a pre-tightening force, and their low bearing capacity.

## Structure and working principles of the proposed high-pressure pre-tightening resin anchor with a compressed grouting body

### Structure

**Fig 2:**
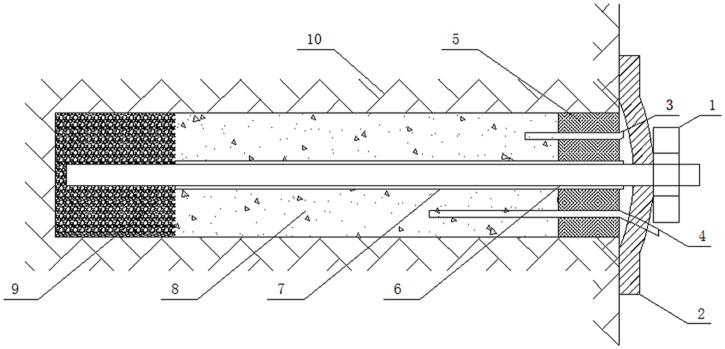
Schematic diagram of the proposed high-pressure pre-tightening resin anchor with a compressed grouting body: 1-nuts, 2-anchor tray, 3-exhaust pipe, 4-grouting pipe, 5-sealing plug, 6-anchor, 7-PVC casing, 8-grouting body, 9-resin body, and 10-surrounding rock.

A conventional grouting anchor has a low pre-tightening force, provides slow initial support, and uses a single anchoring agent. By contrast, the proposed anchor, for which invention patent ZL 2014 1 0124180.4 has been obtained, represents a new type of bolting technology. This anchor combines the rapid coagulation of resin with the excellent bearing capacity of cement mortar after coagulation to provide a rapid, high-strength pre-tightening force during the initial stage and continuous stable anchorage thereafter. The structure of the proposed anchor is shown in Fig 2.

Unlike the bearing mechanism of a conventional grouting anchor, the unique structure of the proposed anchor causes it to display three different working states when it sustains stress during service:

Stage One: Before the grouting body coagulates, only the resin body sustains the pre-tightening force and provides initial support for the surrounding rock.

Stage Two: After the grouting body reaches its design strength (approximately 7 days), the resin body undergoes displacement and deformation because of the tension from the pre-tightening force and squeeze the grouting body. At this time, the grouting body and the resin body jointly serve as a high-strength anchorage for the surrounding rock.

Stage Three: As the resin body sustains an increasing pre-tightening force, it further deforms and debonds from the surrounding rock. However, the anchor does not suffer failure, and the debonded resin body (similar to a bearing plate in a pressure-type anchor) continue to exert pressure on the grouting body, providing continuous and stable anchorage for the surrounding rock via the anchor’s grouting body.

### Manufacturing technology for the proposed anchor

**Fig 3:**
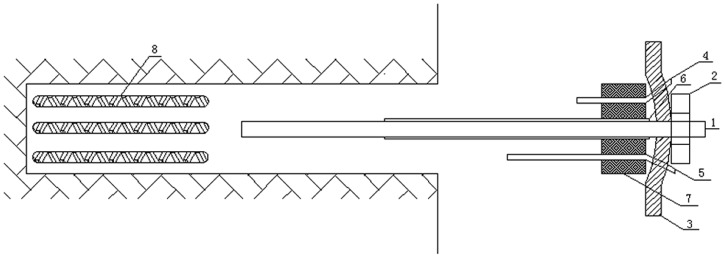
Construction technology diagram for the high-pressure pre-tightening resin anchor with a compressed grouting body: 1-anchor, 2-nut, 3-tray, 4-exhaust pipe, 5-grouting pipe, 6-PVC casing, 7-sealing plug, and 8-resin capsules.

The proposed high-pressure pre-tightening resin anchor with a compressed grouting body differs from conventional anchors not only in its working performance but also in its manufacturing process to ensure its ability to provide increased support for the surrounding rock of a deep roadway. The manufacturing technology required to produce the anchor is illustrated in Fig 3.

The manufacturing steps are as follows:

First, a hole is bored using a jumbolter. Then, the resin capsules are joined end to end and placed into the bored hole while the tray, nut and sealing plug components are assembled, as shown in Fig 3. The inlet valve stem is operated to drive the top of the anchor against the end of the last reel of resin capsules and to slowly send it to the bottom of the hole. The assembled anchor, consisting of components 1–7 in the figure, is pushed into the hole, and the anchor body, driven by the jumbolter, is rotated at a high speed. Then, the resin is stirred for a predetermined length of time. After the resin coagulates (approximately 25 s), a high pre-tightening force is applied to generate tension on the anchor. Next, the anchor is grouted. As the mortar and anchor debond, a pre-tightening force is applied to the anchor again, possibly several times, depending on the on-site support requirements of the surrounding rock.

Compared with the conventional tensile-type anchors that are commonly used in deep roadways, the manufacturing technology for the proposed anchor offers the following advantages:

1. After the resin has been stirred for the predetermined length of time, a pre-tightening force can be immediately applied to the anchor to support the surrounding rock. It is not necessary to wait for the grouting body to reach a certain age first. Therefore, the proposed anchor can prevent the initial deformation of the surrounding rock better than conventional grouting anchors can.
2. The anchor is grouted so that it applies support resistance to the surrounding rock, which can further improve the integrity and self-stability of the surrounding rock.
3. When the grouting body has coagulated and has reached a certain strength, additional pre-tightening force can be applied to the anchor as needed. At this time, the grouting body is in a stressed state, which can further improve the reinforcement of the surrounding rock.

### Analysis of the anchorage performance

Because of its special structure and anchorage principle, the high-pressure pre-tightening resin anchor with a compressed grouting body has the following advantages compared with conventional prestressed end anchor bolting technology and full-length mortar anchor bolting technology:

1. The installation of the free length of the anchor with a PVC casing allows a pre-tightening force to be freely applied to the anchor during grouting.
2. During the initial anchor installation, the resin end anchor structure can provide a rapid and sufficient pre-tightening force for the rock surrounding the roadway. Thus, the anchor achieves active support, and the initial deformation and separation layer of the surrounding rock are controlled to maintain the rock’s integrity and stability.
3. The crevice between the wall of the drill hole and the PVC casing along the free length of the anchor is filled with cement mortar. This mortar guarantees that the grouting body in the free-length section will act as a load-bearing structure, sustaining pressure from the resin anchorage end, under unfavorable displacement or failure in the anchor section. Thus, the mortar prevents the anchor from failing in a weak surrounding rock environment.
4. The grouting body in the free-length section sustains pressure from the resin anchorage end, thereby enabling full utilization of the material properties of the mortar, which has a bearing capacity that is superior to its tensile performance. Therefore, the anchor achieves a higher bearing capacity and better working performance than is possible for an anchor that is solely in tension along its entire length.

## Experimental study of the anchorage performance of the proposed anchor

To further investigate the actual working state and performance of a high-pressure pre-tightening resin anchor with a compressed grouting body, indoor pull-out tests were performed. This study compared and analyzed the strain distributions of the anchors, determined the trends in the bearing capacity for different pre-tightening forces and different values of the elastic modulus of the surrounding rock, and verified the validity of the theoretical analysis.

### Design of the experimental model

Six anchor models were cast for this indoor model experiment: three high-pressure pre-tightening resin anchors with compressed grouting bodies (with surrounding rock types of C20, C30, and C40) and three conventional tensile-type anchors (with the same three surrounding rock types). M30 cement mortar was used for all grouting bodies. A universal testing machine was used to test the strengths and elastic moduli of the three types of surrounding rock and the grouting bodies. The experimental data are listed in Table 1.

**Table 1:** Compressive strengths and elastic moduli of the specimens.

Specimen model	Compressive strength (MPa)	Elastic modulus (GPa)			Mean elastic modulus (GPa)
M30	32.3	11.2	12.7	13.2	12.4
C20	22.8	21.3	24.5	24.9	23.6
C30	34.3	28.8	32.4	30.3	30.5
C40	45.5	35.2	33.4	34.8	34.47

For both anchor types, HRB335 rebar was used for the anchor bodies. Each anchor body had a diameter of 22 mm and uniform dimensions of 1 m × 0.4 m × 0.4 m in length, width and height, respectively. The drilling aperture was 75 mm for all anchors; the anchorage length of the resin for the proposed anchor type was 0.2 m, whereas that of the grouting body was 0.8 m.

### Loading process

**Fig 4:**
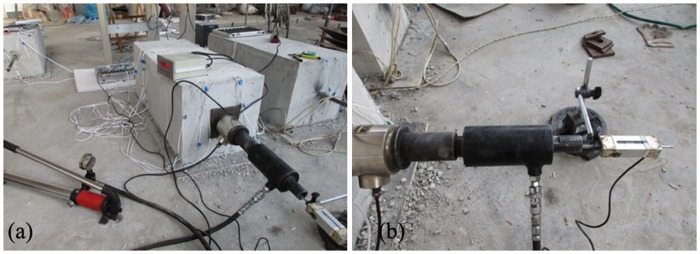
Loading setup for an anchor. (a) Physical model of an anchor pile. (b) Loading devices.

For each tested anchor, a pre-tightening force was applied as a monotonically increasing load. Pre-tightening forces of 20 kN, 40 kN, 60 kN, 80 kN, and 100 kN were successively applied to the anchors to measure the axial strain in the grouting body section; Fig 4 shows the loading setup.

### Analysis of the experimental results

#### The axial strain trends of the anchors under different pre-tightening forces

**Fig 5:**
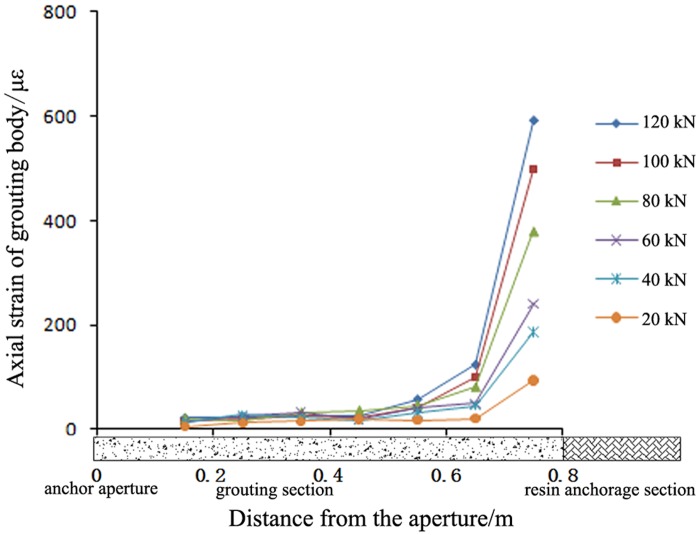
Axial strain in the grouting body of the proposed anchor under different pre-tightening forces. Note: The origin of the coordinate system in Fig 5 is located at the drilling aperture of the anchor.

**Fig 6:**
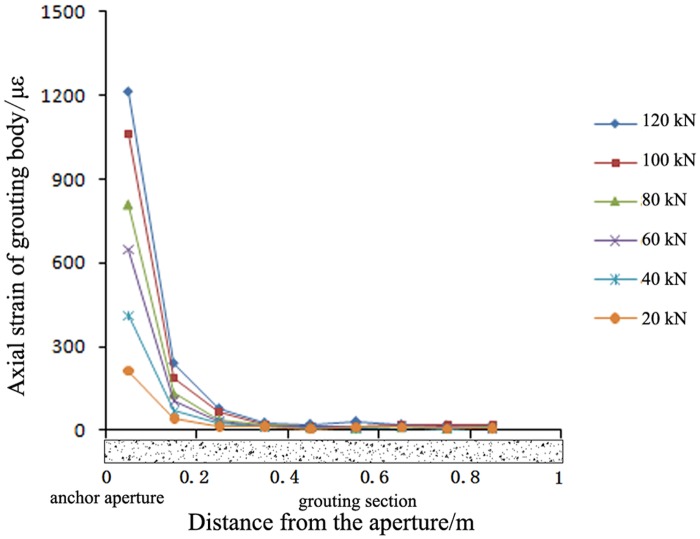
Axial strain in the grouting body of the conventional anchor under different pre-tightening forces. Note: The origin of the coordinate system in Fig 6 is located at the drilling aperture of the anchor.

**Fig 7:**
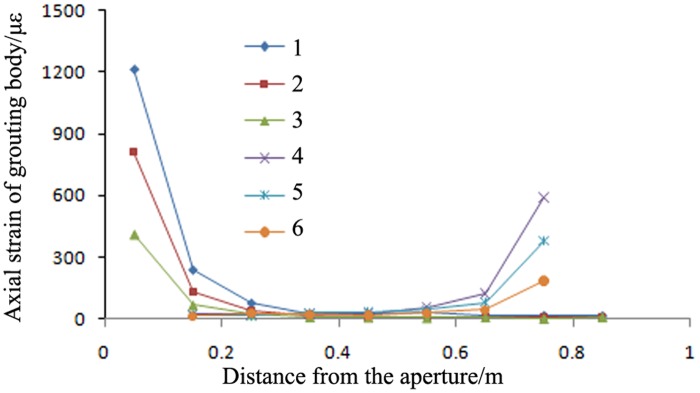
Comparison of the axial strains in the grouting bodies of the proposed anchor and the conventional anchor. 1: conventional anchor, 120 kN; 2: conventional anchor, 80 kN; 3: conventional anchor, 40 kN; 4: proposed anchor, 120 kN; 5: proposed anchor, 80 kN; and 6: proposed anchor, 40 kN.

A high-pressure pre-tightening resin anchor with a compressed grouting body and a conventional tensile-type anchor with surrounding rock of the C30 type were selected for comparison. The axial strain distributions in the grouting bodies of these two anchors are shown in Figs 5–7.

Figs 5–7 show the following:

1. With an increasing pre-tightening force, the axial strain in the proposed anchor increased. However, the length of the strain distribution remained approximately constant. As the pre-tightening force was increased from 20 kN to 120 kN, the peak micro-strain in the anchor grouting body increased from 107 to 591, continuously improving the anchorage for the surrounding rock. The axial strain in the grouting body was always concentrated over a length of 0.4 m from the bearing end and was not distributed evenly along the full length; this result provides further evidence that the full length of the grouting body is not involved in the mechanical stress. Thus, only approximately 0.4 m of the grouting body was involved in the shearing anchorage of the surrounding rock. Therefore, in actual engineering practice, the length of the grouting body can be reduced to lower costs.
2. The strain distributions of the proposed anchor and the conventional anchor were exactly opposite. The axial strain in the grouting body of the proposed anchor was mainly concentrated at the bearing end, anchoring the surrounding rock at the aperture bottom. By contrast, the axial strain in the grouting body of the conventional anchor was mainly concentrated at the opening of the aperture, anchoring the surrounding rock at the aperture opening. Thus, the strain distributions in the grouting body and the anchorage positions for these two anchor types are exactly opposite. Therefore, it is necessary to determine the key position for anchorage and to rationally select the more suitable anchor type based on the actual conditions of the surrounding rock.
3. Under the same pre-tightening force, the axial strain in the grouting body of the proposed anchor was smaller than that in the grouting body of the conventional anchor.

As the pre-tightening force was increased from 20 kN to 120 kN, the peak micro-strain in the conventional anchor increased from 212 to 1215. However, for the proposed anchor, the peak micro-strain increased only from 107 to 591. Thus, under a pre-tightening force of the same magnitude, the peak micro-strain in the proposed anchor was always smaller than that in the conventional anchor. Therefore, an anchor of the proposed type is less likely to fail than a conventional anchor, and the proposed anchor type has a higher bearing capacity.

#### The axial strain trends of the anchors for different values of the elastic modulus of the surrounding rock

**Fig 8:**
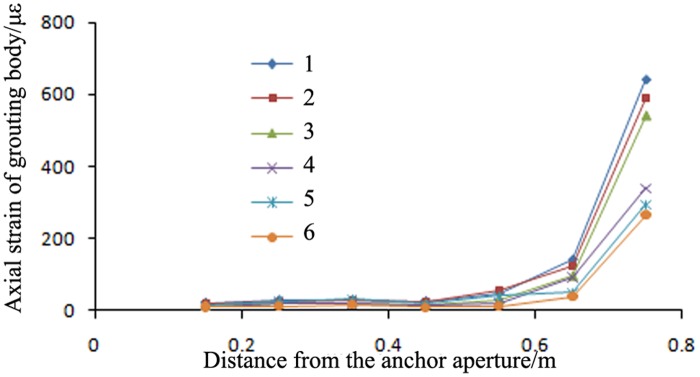
Axial strains in the grouting bodies of high-pressure pre-tightening resin anchors with compressed grouting bodies in surrounding rock with different elastic moduli. 1: pull-out force of 120 kN and C20 surrounding rock; 2: pull-out force of 120 kN and C30 surrounding rock; 3: pull-out force of 120 kN and C40 surrounding rock; 4: pull-out force of 60 kN and C20 surrounding rock; 5: pull-out force of 60 kN and C30 surrounding rock; and 6: pull-out force of 60 kN and C40 surrounding rock.

**Fig 9:**
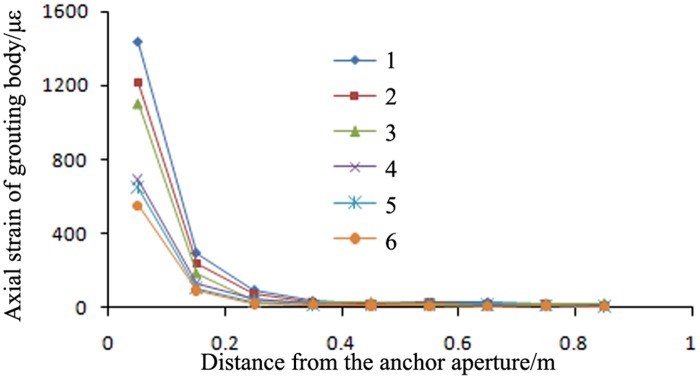
Axial strains in the grouting bodies of conventional tensile-type anchors in surrounding rock with different elastic moduli. 1: pull-out force of 120 kN and C20 surrounding rock; 2: pull-out force of 120 kN and C30 surrounding rock; 3: pull-out force of 120 kN and C40 surrounding rock; 4: pull-out force of 60 kN and C20 surrounding rock; 5: pull-out force of 60 kN and C30 surrounding rock; and 6: pull-out force of 60 kN and C40 surrounding rock.

The trends of the axial strains in the grouting bodies of a high-pressure pre-tightening resin anchor with a compressed grouting body and a conventional tensile-type anchor in surrounding rock with three different elastic moduli (i.e., rock types C20, C30 and C40) are shown in Figs 8 and 9.

Figs 8 and 9 show the following:

1. As the elastic modulus of the surrounding rock increased, the peak axial strain in the anchors of the proposed type monotonically decreased; however, the length of the axial strain distribution remained approximately constant. When the surrounding rock type changed from C20 to C40 (and the elastic modulus increased from 2.36 × 10^10^ Pa to 3.45 × 10^10^ Pa), the peak axial micro-strain in the grouting bodies of the proposed anchors decreased from 642 to 541. However, because of the small increase in the elastic modulus of the surrounding rock, the length of the anchor grouting body over which the axial strain was distributed remained approximately constant and was always concentrated within 0.4 m from the bearing end. Therefore, regardless of the type of the surrounding rock and its elastic modulus, the length of the grouting body can be reduced to 0.4 m to decrease engineering costs.
2. Regardless of the surrounding rock type, the strain sustained by the proposed anchors was always smaller than that sustained by the conventional anchors. When the surrounding rock type changed from C20 to C40, the peak axial micro-strains in the conventional anchors decreased from 1433 to 1105; these values were larger than the peak axial micro-strains in the proposed anchors, which decreased from 642 to 541. Therefore, regardless of the surrounding rock type, the ultimate bearing capacities of the anchors of the proposed type were always greater than those of the conventional anchors.

#### Comparative analysis of the displacement at the pull-out end of the anchor

**Fig 10:**
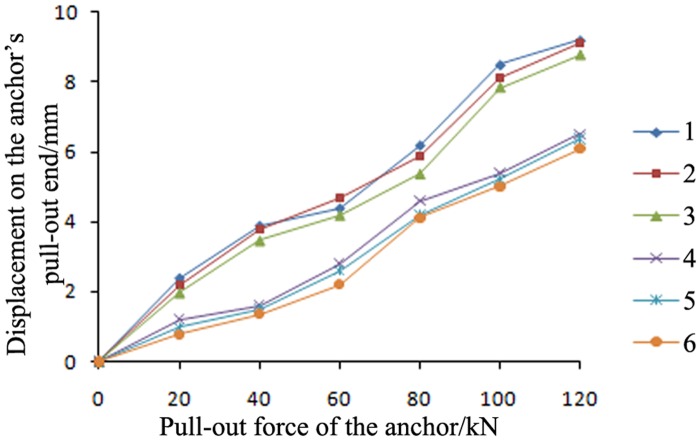
Actual displacement at the pull-out end of the anchor. 1: conventional anchor, C20; 2: conventional anchor, C30; 3: conventional anchor, C40; 4: proposed anchor, C20; 5: proposed anchor, C30; and 6: proposed anchor, C40.

Fig 10 shows the following:

1. Under the same pre-tightening force, the displacement of the grouting body was smaller for the proposed anchor than for the conventional anchor. In addition, the bearing capacity of the grouting body was greater for the proposed anchor. The displacement *S* at the tension end of the proposed anchor was equal to the grouting body displacement *S*_*1*_ plus the anchor body displacement *S*_*2*_.
  The 0.8-m-long grouting bodies of the anchors of the proposed type were all free and unbonded. Therefore, under the same pre-tightening force, the anchor body displacement *S*_*2*_ of the proposed anchor was greater than that of the conventional anchor, *S*^*’*^_*2*_. Fig 10 shows that the displacement *S* at the tension end of the proposed anchor (with a value between 6.09 and 6.5 mm) was smaller than the displacement *S’* at the tension end of the conventional anchor (between 8.77 and 9.2 mm). Therefore, the displacement of the grouting body of the proposed anchor, *S*_*1*_, was also smaller than that of the conventional anchor, *S*^*’*^_*1*_. In addition, the proposed anchor had a larger ultimate bearing capacity.
2. Throughout the entire tensioning process, both the proposed and conventional anchors were undergoing elastic deformation (tension failure and damage occurred in both). The increase in the displacement at the tension end was fairly constant, and the bearing capacities of both anchors in hard rock were greater than 120 kN.

## Conclusions

The following conclusions can be drawn from the current study:

1. The proposed high-pressure pre-tightening resin anchor with a compressed grouting body is a new bolting technology that provides rapid, high-strength pre-tightening for initial support as well as continuous and stable anchorage. This new anchor combines the rapid coagulation of resin with the excellent bearing capacity of cement mortar after coagulation.
2. A high-pressure pre-tightening resin anchor with a compressed grouting body undergoes three loading stages. In the first stage, the resin body provides the initial anchorage for the surrounding rock. In the second stage, the resin body and the grouting body jointly offer high-strength anchorage for the surrounding rock. In the third stage, after the resin body debonds, the grouting body provides continuous and steady anchorage for the surrounding rock.
3. A high-pressure pre-tightening resin anchor with a compressed grouting body mainly provides anchorage at the bottom of the aperture in the surrounding rock, whereas a conventional tensile-type anchor mainly provides anchorage at the opening of the aperture in the surrounding rock. Thus, the anchorage positions for these two anchor types are exactly opposite.
4. Regardless of the type of the surrounding rock and its elastic modulus, the ultimate bearing capacity of a high-pressure pre-tightening resin anchor with a compressed grouting body is always higher than that of a conventional tensile-type anchor. Of the entire grouting body of the proposed anchor, only approximately 0.4 m is involved in the anchorage of the surrounding rock; therefore, the length of the grouting body can be reduced to 0.4 m to decrease engineering costs.

## References

[1] Xie S, Li E, Li S, Wang JG, He CC, Yang YF. Surrounding rock control mechanism of deep coal roadways and its application. Int J Min Sci Technol. 2015;25: 429–434.

[2] Yang Xj, Pang JW, Liu DM, Liu Y, Tian YH, Ma J, et al Deformation mechanism of roadways in deep soft rock at Hegang Xing’an Coal Mine. Int J Min Sci Technol. 2013;23: 307–312.

[3] Meng Q, Han L, Chen Y, Fan J, Wen S, Yu L, et al Influence of dynamic pressure on deep underground soft rock roadway support and its application. Int J Min Sci Technol. 2016.

[4] Guo Z, Yang X, Bai Y, Zhou F, Li EQ. A study of support strategies in deep soft rock: The horsehead crossing roadway in Daqiang Coal Mine. Int J Min Sci Technol. 2012;22: 665–667.

[5] Kang HP. Development and application of pre-stress anchored bolt supporting technology in coal mine. Coal technol. 2011;16: 25–30.

[6] Xie S, Li E, Li S, Wang J, He C, Yang Y. Surrounding rock control mechanism of deep coal roadways and its application. Int J Min Sci Technol. 2015;25: 429–434.

[7] Pengfei J, Jian L, Bin H. The Deformation Mechanism and Support Methods of the Water-Bearing Soft Rock Roadway. Physical and Numerical Simulation of Geotechnical Engineering. 2016;22: 55.

[8] Yan H, Weng M, Feng R, Li W. Layout and support design of a coal roadway in ultra-close multiple-seams. J Central South Univ. 2015;22: 4385–4395.

[9] Meng Q, Han L, Sun J, Min F, Feng W, Zhou X. Experimental study on the bolt—cable combined supporting technology for the extraction roadways in weakly cemented strata. Int J Min Sci Technol. 2015;25: 113–119.

[10] Liu QS, Lu CB, Liu B, Liu XW. Research on the grouting diffusion mechanism and its application of grouting reinforcement in deep roadway. J Min Safety Engineer. 2014;31: 333–339.

[11] Yang RS, Xue HJ, Guo DM, Li TT, He TY, Gao ZM. Grouting reinforcement support technology of soft and weak coal sides in large section roadway. Coal Sci Technol. 2014;42: 1–4.

[12] Kang HP, Feng ZQ. Status and development tendency of roadway grouting reinforcement technology in coal mine. Coal Min Technol. 2013;18: 1–7.

[13] Zhang K, Zhang G, Hou R, Wu Y, Zhou H. Stress evolution in roadway rock bolts during mining in a fully mechanized longwall face, and an evaluation of rock bolt support design. Rock Mechanics & Rock Engineering. 2015;48: 333–344.

[14] Li H, Liu W J, Qiao W G. Study on High Prestressed Anchor Beam Supporting Optimization Technology in Deep Roadway. Appli Mech Mater. 2013;353: 1675–1679.

[15] Li S, Wang H, Wang Q, Jiang B, Wang F, Guo N, et al Failure mechanism of bolting support and high-strength bolt-grouting technology for deep and soft surrounding rock with high stress. J Central South Univ. 2016;23: 440–448.

[16] Huang Y, Wang L, Zhang J. Reinforcement Mechanism and Slurry Diffusion Law of Deep-shallow Coupled Fully Bolting-grouting in Deep Broken Roadway. Electr J Geotechnical Engineer. 2014;19: 6319–6330.

[17] Kang H. Support technologies for deep and complex roadways in underground coal mines: a review. Inter J Coal Sci Tech. 2014;1: 261–277.

